# Toll-like receptor 4 mediates blood-brain barrier permeability and disease in C3H mice during Venezuelan equine encephalitis virus infection: Venezuelan equine encephalitis virus and toll-like receptor 4

**DOI:** 10.1080/21505594.2020.1870834

**Published:** 2021-01-25

**Authors:** Bradley S. Hollidge, Courtney A. Cohen, Justice Akuoku Frimpong, Catherine V. Badger, John M. Dye, Connie S. Schmaljohn

**Affiliations:** a Virology Division, United States Army Medical Research Institute of Infectious Diseases, Fort Detrick, Maryland, USA; b Headquarters Division, United States Army Medical Research Institute of Infectious Diseases, Fort Detrick, Maryland, USA; c REGENXBIO, Inc., Rockville, Maryland, USA; d Immunodiagnostics Department, Biological Defense Research Directorate, Naval Medical Research Center, Fort Detrick, Maryland, USA; e Integrated Research Facility, National Institute of Allergy and Infectious Diseases, National Institute of Health, Fort Detrick, Maryland, USA

**Keywords:** Venezuelan equine encephalitis virus, blood-brain barrier, toll-like receptor 4, veev, tlr4

## Abstract

Venezuelan equine encephalitis virus (VEEV) is an encephalitic alphavirus that can cause debilitating, acute febrile illness and potentially result in encephalitis. Currently, there are no FDA-licensed vaccines or specific therapeutics for VEEV. Previous studies have demonstrated that VEEV infection results in increased blood-brain barrier (BBB) permeability that is mediated by matrix metalloproteinases (MMPs). Furthermore, after subarachnoid hemorrhage in mice, MMP-9 is upregulated in the brain and mediates BBB permeability in a toll-like receptor 4 (TLR4)-dependent manner. Here, we demonstrate that disease in C3H mice during VEEV TC-83 infection is dependent on TLR4 because intranasal infection of C3H/HeN (TLR4*^WT^*) mice with VEEV TC-83 resulted in mortality as opposed to survival of TLR4-defective C3H/HeJ (TLR4*^mut^*) mice. In addition, BBB permeability was induced to a lesser extent in TLR4*^mut^* mice compared with TLR4*^WT^* mice during VEEV TC-83 infection as determined by sodium fluorescein and fluorescently-conjugated dextran extravasation. Moreover, MMP-9, MMP-2, ICAM-1, CCL2 and IFN-γ were all induced to significantly lower levels in the brains of infected TLR4*^mut^* mice compared with infected TLR4*^WT^* mice despite the absence of significantly different viral titers or immune cell populations in the brains of infected TLR4*^WT^* and TLR4*^mut^* mice. These data demonstrate the critical role of TLR4 in mediating BBB permeability and disease in C3H mice during VEEV TC-83 infection, which suggests that TLR4 is a potential target for the development of therapeutics for VEEV.

## Introduction

Venezuelan equine encephalitis virus (VEEV) is an arthropod-borne virus (arbovirus) in the *Alphavirus* genus of the *Togaviridae* family and is one of the New World alphaviruses, which are mostly encephalitogenic [1,2]. This single-stranded, positive-sense RNA virus has sporadically caused widespread epidemics and epizootics in the Western Hemisphere as it is highly pathogenic for both humans and equines [1,3]. The incubation period of VEEV in humans is approximately 1–8 days after infection and VEEV infection in humans typically results in an asymptomatic infection or an acute, febrile illness with malaise, severe retro-orbital or occipital headaches, myalgia, nausea and vomiting as common features [3,4]. Even though the case-fatality rate is low (<1%), disease caused by VEEV infection can also progress to the central nervous system (CNS) in 4–14% of human cases resulting in neurologic disease [3]. The encephalitic disease is variable in severity ranging from somnolence and mild confusion to seizures, ataxia, paralysis and coma [5]. As a result of it producing incapacitating or lethal infections and being infectious by aerosols as well as its ease of being grown to high titers in elementary cell culture systems, VEEV represents a significant potential biologic threat [6]. VEEV is classified as a category B priority biodefense agent by both the Centers for Disease Control and Prevention and the National Institute of Allergy and Infectious Diseases. Currently, there are no approved vaccines or specific therapies for VEEV. As evidenced by the occurrence of laboratory-acquired infections involving aerosols, aerosolized VEEV is highly infectious to humans, potentially resulting in higher mortality than natural infections [7,8].

In murine models, VEEV rapidly enters the CNS following aerosol or intranasal exposure via the olfactory neuroepithelium and olfactory bulb where it initially replicates [9–11]. In addition, intranasal inoculation of VEEV replicon particles, which undergo only a single round of replication with no propagation or spread, induces an inflammatory cascade and an increase in blood-brain barrier (BBB) permeability [12]. Despite the restriction of viral RNA to the olfactory bulb, the increased BBB permeability is disseminated throughout the brain suggesting the propagation of a signal in the brain that induces an increase in BBB permeability. Furthermore, treatment of VEEV-infected mice with a broad-spectrum matrix metalloproteinase (MMP) inhibitor reduced the induction of BBB permeability during infection, delayed onset of disease and reduced mortality [12]. These findings complement studies of West Nile virus (WNV) and Rift Valley fever virus implicating MMP-9 in mediating BBB permeability [13,14] and demonstrate that increased BBB permeability is a critical step in the neuropathogenesis of these encephalitic viruses.

Innate immune signaling within the CNS can alter BBB permeability in the context of infection [15] or injury (e.g. cerebral ischemia) [16] through the induction of cytokines, chemokines and neurovascular proteases including MMPs. In particular, upregulation of MMP-9 results in increased BBB permeability [12,14,16–19], vasogenic edema [20,21] and hemorrhage [22,23]. Recently, inhibition of toll-like receptor 4 (TLR4) was found to prevent the induction of increased BBB permeability and suppress the upregulation of MMP-9 after subarachnoid hemorrhage in mice [16]. Additionally, upregulation of MMP-9 has also been reported to be dependent on TLR2 in intracerebral hemorrhage models [24]. Moreover, high-mobility group box 1 (HMGB1) is released resulting in TLR4-dependent MMP-9 upregulation in the brain following cerebral ischemia in mice [25]. Taken together, these studies demonstrate a role for innate immune signaling in the induction of BBB permeability.

In addition, the innate immune system in the CNS provides potential targets for treatment of VEEV since modulation of the innate immune system can alter the lethality of virus infections. For example, TLR4-knockout mice infected with Japanese encephalitis virus (JEV) show reduced CNS inflammation, viral RNA, leukocyte infiltration, proinflammatory cytokine expression and, importantly, increased resistance to lethal infection [26]. In addition, TLR2 mediates the induction of inflammatory cytokines and mortality in mice infected with herpes simplex virus 1 [27]. Therefore, TLRs play a central role in the pathogenesis of neurotropic viruses.

Here, we investigated the role of TLR4 in the induction of BBB permeability and disease in mice during VEEV infection using the C3H/HeN model of VEEV infection with the TC-83 vaccine strain, which causes encephalitis and death in C3H/HeN (TLR4*^WT^*) mice when intranasally instilled, in parallel with TLR4-defective C3H/HeJ (TLR4*^mut^*) mice.

## Materials and methods

### Virus

The live-attenuated VEEV vaccine TC-83 (NDBR-102, lot 4, run 3; National Drug Company, Swiftwater, PA, USA) was passaged twice in BHK cells before sucrose purification.

### Ethics statement

Research was conducted under an Institutional Animal Care and Use Committee (IACUC)-approved protocol in compliance with the Animal Welfare Act, PHS Policy, and other Federal statutes and regulations relating to animals and experiments involving animals. The facility where this research was conducted is accredited by the AAALAC International and adheres to principles stated in the Guide for the Care and Use of Laboratory Animals, National Research Council, 2011.

### Animal experiments

C3H/HeN (TLR4*^WT^*) mice were purchased from Jackson Laboratories and C3H/HeJ (TLR4*^mut^*) mice were purchased from Charles River Laboratories. In all studies, 6–8 week-old female mice were intranasally instilled (20 µL per nostril) with 2 × 10^7^ plaque-forming units (PFU) of VEEV TC-83 in 40 µL phosphate-buffered saline (PBS) or 40 µL PBS alone under light isoflurane anesthesia. Mice were weighed daily and monitored 1–3 times per day for survival and signs of disease.

#### BBB permeability assessment

To assess BBB permeability, mice were injected intraperitoneally with 10 mg of sodium fluorescein salt (Sigma-Aldrich) or 400 µg of 3kDa-dextran conjugated to Alexa Fluor 680 (Invitrogen) in sterile PBS. Approximately 40 min after this injection, mice were anesthetized with ketamine-acepromazine-xylazine for approximately 5 min until unresponsive. Blood was collected by a terminal cardiac bleed followed by cardiac perfusion with PBS. Serum was isolated using a serum separator and was stored at −80°C for subsequent analysis. Brains were harvested and stored at −80°C until homogenization. One hemisphere of each brain from mice that were administered 3kDa-dextran conjugated to Alexa Fluor 680 was fixed in formalin and Alexa Fluor 680 fluorescence was imaged using a Licor Odyssey CLx (Li-cor Biosciences).

Each brain region was homogenized in 1 mL of PBS using GentleMACS M tubes (Miltenyi Biotech). Sodium fluorescein and Alexa Fluor 680 fluorescence in brain homogenates were measured undiluted or diluted in PBS. To measure sodium fluorescein in the brain, homogenates were diluted 1:1 with 2% trichloroacetic acid (Thermo Scientific) to precipitate protein and incubated at 4°C overnight, centrifuged at 3,000 rpm for 10 min, and then supernatants were diluted 1:1 with borate buffer (0.05 M, pH 11; Ricca Chemical). Sample fluorescence of fluorescein (excitation at 480 nm; emission at 538) or Alexa Fluor 680 (excitation at 679; emission 702) was measured using a Tecan Infinite M1000 Pro microplate reader. The amount of fluorescein in each tissue sample and serum sample was determined using standards ranging from 3.7 µg to 3750 µg in 0.025 M borate buffer and 1% thichloroacetic acid. The tissue fluorescein was normalized to tissue weight and the BBB permeability was determined as the ratio of the amount of sodium fluorescein measured in the brain region to the amount measured in the serum for each mouse. The relative BBB permeability is expressed as the BBB permeability of infected mice normalized to the BBB permeability of uninfected mice of the same genotype.

#### Plaque assays

In six-well plates, Vero 76 cells were plated at a density of 3 × 10^5^ cells/well and incubated for 48 h at 37°C, 5% CO_2_. Serial dilutions of tissue homogenates were made in 1x Minimum Essential Medium with 1% penicillin/streptomycin and 10% (v/v) heat-inactivated fetal bovine serum (HI-FBS). The serially-diluted homogenates were incubated on the Vero cell monolayers for 1 h at 37°C, 5% CO_2_ with gentle rocking every 15 min. Subsequently, cells were overlaid with 2 ml of a 1:1 mixture of 1% (w/v) agarose and 2X Basal Medium Eagle with Earle’s Salts (EBME) containing 10% HI-FBS, 2% penicillin/streptomycin, 50 µg/mL gentamicin, and 2.5 µg/mL Fungizone (amphotericin B). The plates were incubated overnight with the 0.5% agarose/EBME overlay at 37°C, 5% CO_2_. After approximately 24 h, 2 ml of a secondary 0.5%/EBME overlay with 4% neutral red was added to each well and then incubated overnight at 37°C, 5% CO_2_. The visualized plaques were counted.

#### Magpix multiplex assays

The concentration of CCL-2, ICAM-1, MMP-2, MMP-9, IL-2, IL-4, IL-6, TNF-α and IFN-γ in the brain homogenates were determined with multiplex magnetic bead-based immunoassays (mouse magnetic Luminex assay [R&D Systems] – CCL-2, ICAM-1, MMP-2 and MMP-9; high sensitivity 5-plex mouse ProcartaPlex panel [Invitrogen] – IL-2, IL-4, IL-6, TNF-α and IFN-γ) according to the manufacturers’ instructions. The samples were analyzed using a MAGPIX analyzer (Luminex Inc.).

#### Serum IFN-γ ELISA

IFN-γ concentrations were determined using a Quantikine mouse IFN-γ ELISA (R&D Systems) and according to the manufacturer’s instructions.

#### Flow cytometry

Brain homogenates were prepared as previously described [28]. Briefly, tissue was incubated at 37°C for 45 min in digestion buffer (1% FBS, 1 mg/ml collagenase D, 0.1 mg/ml DNase in RPMI) and then homogenized using the GentleMACS Dissociator system (Miltenyi Biotech). Mononuclear cell suspensions were isolated by filtering homogenate through a 70 µm cell strainer and then centrifuging on a 30/70% Percoll gradient for 30 min at 4°C. Immune cells were collected from the interface, stained with amine-reactive Aqua fixable dye (Thermo Fisher) to exclude dead cells, and nonspecific staining was restricted by using rat anti-mouse CD16/CD32 (BD Biosciences). Cells were surface stained for conventional immune cell markers, and then fixed, permeabilized and stained for intracellular cytokine analysis. Cell types were defined as follows: Resting microglia (CD45^low^CD11b^low^), activated microglia (CD45^high^CD11b^low^CD11c^−^Ly6C^−^Ly6G^−^), T cells (CD45^high^CD3^+^), B cells (CD45^high^CD19^+^), infiltrating macrophages (CD45^high^CD11b^+^MHCcII^+^Ly6C^high^), inflammatory monocytes (CD45^high^CD11b^+^CD11c^−^MHCcII^−^Ly6C^high^Ly6G^−^SSC^high^), non-classical monocytes (CD45^high^CD11b^+^CD11c^−^MHCcII^−^Ly6C^+^Ly6G^−^SSC^high^), polymorphonuclear neutrophils (CD45^high^CD11b^+^CD11c^−^MHCcII^−^Ly6C^+^Ly6G^+^), natural killer cells (CD45^high^CD11b^low^CD11c^−^MHCcII^−^CD49b^+^SSC^low^) and dendritic cells (CD45^high^CD11b^±^CD11c^+^). Samples were acquired on a Fortessa X20 flow cytometer (BD Biosciences) with FACS Diva software, and analyzed using Flowjo (Treestar, version X).

#### Statistical analysis

Kaplan-Meier survival curves were analyzed by the log-rank test. The statistical significance of all other data was evaluated by unpaired two-tailed Student’s t-tests or Mann-Whitney tests. A *p*-value of <0.05 was considered significant. All data are expressed as the mean ± standard error of the mean (SEM). Statistical analysis was performed using GraphPad Prism 8 software. Data shown have been confirmed in independent experiments (data not shown) with the exception of flow cytometry experiments.

## Results

### TLR4^mut^ mice are less susceptible to disease during VEEV TC-83 infection

**Figure 1.**
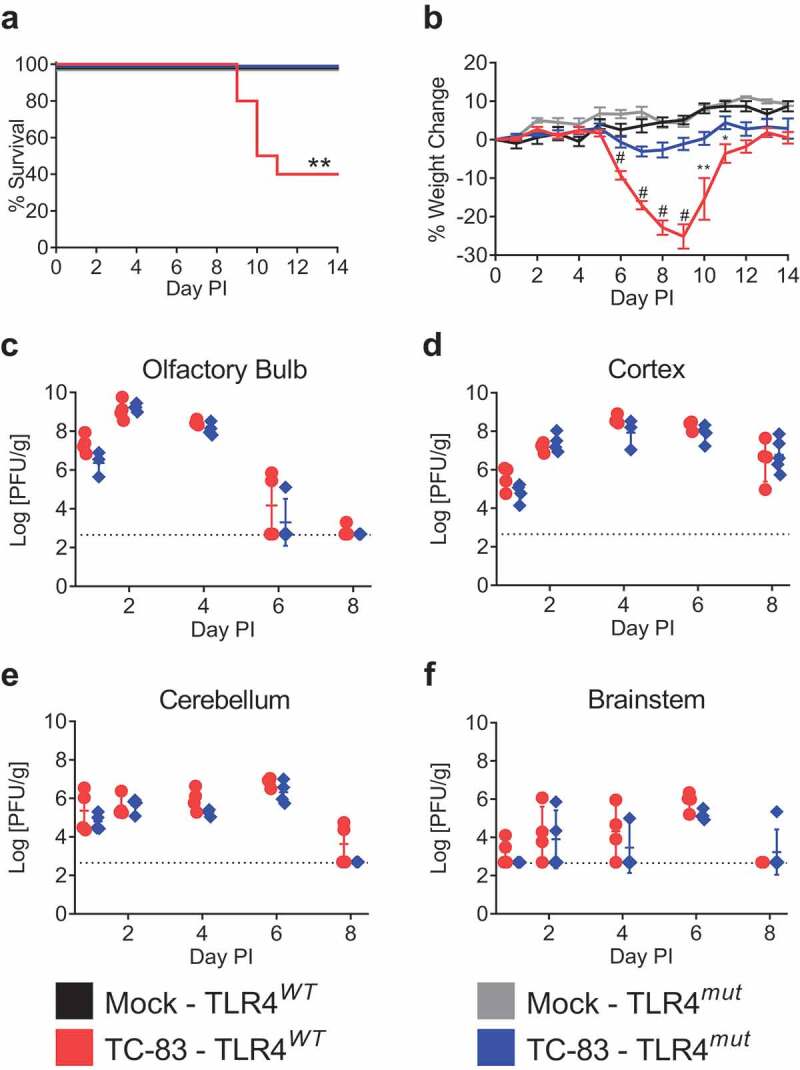
**Survival, weight change and brain viral titers in TLR4*^WT^* mice and TLR4*^mut^* mice during VEEV TC-83 infection**. TLR4*^WT^* mice and TLR4*^mut^* mice were intranasally instilled with 2 × 10^7^ PFU of VEEV TC-83 (*n* = 10 mice per group) or PBS (*n* = 5 mice per group). Mice were monitored for survival (a) and weight change (b). The viral titers in the brains of TLR4*^WT^* mice and TLR4*^mut^* mice were determined by plaque assay from the olfactory bulb (c), cortex (d), cerebellum (e) and brainstem (E). Data are presented as mean ± SEM (*n* = 3–5 mice per group). The dotted line represents the limit of detection for the lowest dilution tested. Statistically significant differences between infected TLR4*^WT^* and TLR4*^mut^* are indicated as *, *p* < 0.05; **, *p* < 0.01 and #, *p* < 0.0001. Kaplan-Meier survival curves were analyzed by the log-rank test. The statistical significance of all other data was evaluated by unpaired two-tailed Student’s t-tests or Mann-Whitney tests

Previous studies have shown that VEEV replication in the CNS precedes the increase in BBB permeability and that inhibition of MMPs decreases the induced BBB permeability as well as increases survival times and survival rates of mice challenged subcutaneously [12]. In addition, MMP-9 is upregulated in a number of neurologic diseases [12,14,17,19–22,24], which can be mediated by TLR4 [16,25]. Moreover, C3H/HeN (TLR4*^WT^*) mice are susceptible to intranasal exposure of VEEV TC-83 and develop encephalitic disease [11,29,30], unlike other inbred mouse strains. Conveniently, the C3H/HeJ substrain (TLR4*^mut^*) of C3H mice, which was characterized as LPS hyporesponsive, has a single point mutation in the *Tlr4* gene that results in a defective TLR4 protein [31]. Therefore, to determine if TLR4 alters the susceptibility of C3H mice to intranasal exposure of VEEV TC-83, TLR4*^WT^* mice and TLR4*^mut^* mice were intranasally instilled with 2 × 10^7^ PFU and were subsequently monitored for disease and survival. As expected, a majority of the TLR4*^WT^* mice that were intranasally infected with VEEV TC-83 succumbed to the infection with a mortality rate of 60% and a mean time-to-death of 9.8 days (Figure 1a). These TLR4*^WT^* mice began losing weight at day 6 post-infection (PI), which continued until day 9 (−25.1 ± 3.2% baseline weight; Figure 1b), at which point the mice began to succumb to disease. The surviving TLR4*^WT^* mice lost an average of 19.7 ± 2.1% of their baseline bodyweight during peak disease. In contrast to the TLR4*^WT^* mice, 100% of the TLR4*^mut^* mice survived VEEV TC-83 infection (Figure 1a; *p* = 0.0041, log-rank test). Moreover, these mice began to lose weight at day 6 PI (−0.6 ± 1.4% baseline weight), peaked at day 7 (−3.1 ± 1.3% baseline weight), and then began to regain weight (Figure 1b). Other than weight loss, the TLR4*^mut^* mice showed no overt signs of disease.

Despite the significant differences in mortality and disease, there were no significant differences in the viral loads in the brains at days 1, 2, 4, 6 or 8 PI between the two mouse substrains (Figure 1c-f). These data indicate that the encephalitic disease observed in mice infected with VEEV TC-83 is not exclusively dependent on viral load.

### TLR4 mediates BBB permeability during VEEV TC-83 infection

**Figure 2.**
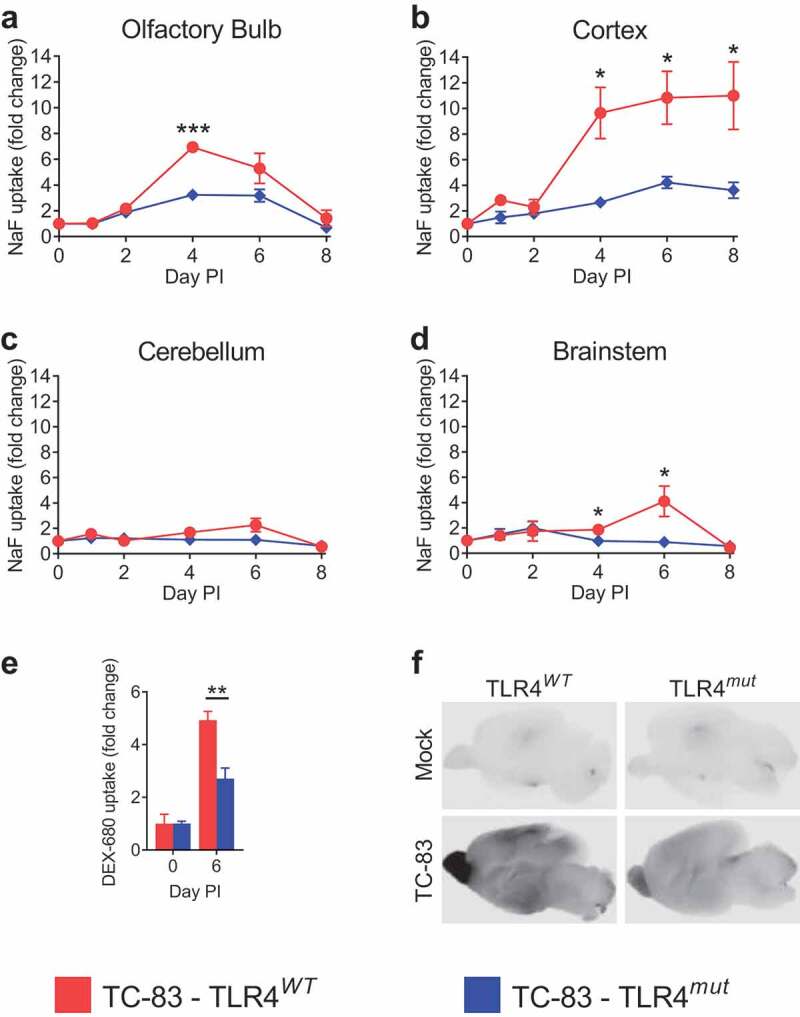
**BBB permeability in TLR4*^WT^* mice and TLR4*^mut^* mice during VEEV TC-83 infection**. The changes in BBB permeability in VEEV TC-83-infected TLR4*^WT^* mice and TLR4*^mut^* mice were assessed by measuring the uptake of NaF in the olfactory bulbs (a), cortexes (b), cerebellums (c) and brainstems (d). The uptake of DEX-680 in the cortexes of TLR4*^WT^* mice and TLR4*^mut^* mice were determined at day 6 PI (e) and visualized by imaging on a near-infrared imager (f). Tissue uptake of NaF or DEX-680 for each brain region was the ratio of the amount of fluorophore in each region to the amount of fluorophore in the serum for each mouse. NaF and DEX-680 uptake is expressed as the fold change over uninfected control mice of the same genotype. Higher fluorophore uptake represents increased BBB permeability. Mock-infected (PBS-instilled) mice are represented as Day 0 PI. Data are presented as mean ± SEM (*n* = 3–5 mice per group). Statistically significant differences are indicated as *, *p* < 0.05; **, *p* < 0.01. Statistical significance was evaluated by unpaired two-tailed Student’s t-tests or Mann-Whitney tests

To determine the dependence of increased BBB permeability during VEEV TC-83 infection, BBB integrity was measured by the extravasation of sodium fluorescein (NaF) in different brain regions. At days 1, 2, 4, 6 and 8 PI, TLR4*^WT^* mice and TLR4*^mut^* mice were intraperitoneally injected with NaF. After 45 min, sera and perfused brains were harvested and the brains were assessed for BBB permeability by measuring the amount of NaF in the brains relative to the sera. In the olfactory bulb, BBB permeability began to increase in the TLR4*^WT^* mice at day 2 PI and continued to increase until day 4 PI, after which BBB permeability began to decrease and return to baseline at day 8 PI. However, the BBB permeability in the olfactory bulbs at day 4 PI in TLR4*^mut^* mice was induced to a lower level compared with infected TLR4*^WT^* mice (Figure 2a). More strikingly, the VEEV TC-83-induced BBB permeability in the cortexes of TLR4*^mut^* mice was induced to significantly lesser extents at days 4, 6 and 8 PI compared with infected TLR4*^WT^* mice (Figure 2b). The induction of BBB permeability in the cerebellums (Figure 2c) and brainstems (Figure 2d) of both strains of mice was lower compared to the permeabilities of the olfactory bulbs and cortexes. To confirm the role of TLR4 in BBB permeability during VEEV TC-83 infection found by NaF extravasation, TLR4*^WT^* and TLR4*^mut^* mice were injected with 3kDa-dextran conjugated to Alexa Fluor 680 (DEX-680) at day 6 PI. As shown in (Figure 2(e**,f))**, BBB permeability was induced to a significantly lesser extent in the infected TLR4*^mut^* mice compared with the infected TLR4*^WT^* mice. These data demonstrate that TLR4 mediates increased BBB permeability during VEEV TC-83 infection.

### Impaired TLR4 signaling reduces the upregulation of BBB permeability-related proteins during VEEV TC-83 infection

**Figure 3.**
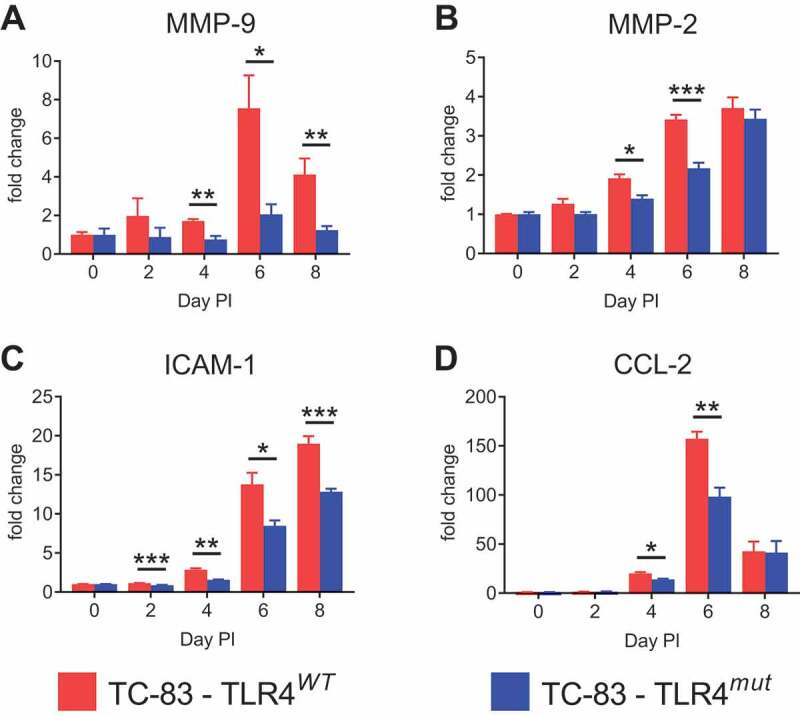
**Induction of BBB permeability-associated proteins in the cortexes of TLR4*^WT^* mice and TLR4*^mut^* mice during VEEV TC-83 infection**. The inductions of MMP-9 (a), MMP-2 (b), ICAM-1 (c) and CCL-2 (d) during VEEV TC-83 infection were assessed in cortical tissue from TLR4*^WT^* mice and TLR4*^mut^* mice. The fold change of these proteins is expressed as the ratio of the amount of protein in infected mice relative to uninfected mice of the same genotype. Mock-infected (PBS-instilled) mice are represented as Day 0 PI. Data are presented as mean ± SEM (*n* = 3–5 mice per group). Statistically significant differences are indicated as *, *p* < 0.05; **, *p* < 0.01; ***, *p* < 0.001. Statistical significance was evaluated by unpaired two-tailed Student’s t-tests or Mann-Whitney tests

Several proteins involved in BBB permeability were measured in the cortexes of TLR4*^WT^* and TLR4*^mut^* mice infected with VEEV TC-83. MMP-9 was of particular interest because its expression can be dependent on TLR4 in the brain [16,25] and it can disrupt the BBB by degrading tight junction proteins and basal lamina proteins. Furthermore, MMP-9 has been shown to mediate increased BBB permeability in neurologic diseases, including during WNV infection [14] and VEEV infection of mice [12]. As expected, there was a significant upregulation of MMP-9 in the cortexes of TC-83-infected TLR4*^WT^* mice at days 4, 6 and 8 PI, which coincides with increased cortical BBB permeability, compared with mock-infected TLR4*^WT^* mice. However, there was no significant upregulation in MMP-9 in the cortexes of TC-83-infected TLR4*^mut^* mice compared with mock-infected TLR4*^mut^* mice. Furthermore, MMP-9 was induced to significantly lesser extents in the cortexes of infected TLR4*^mut^* mice compared with the TLR4*^WT^* mice (Figure 3a). In addition, there were significant inductions of MMP-2 in the brains of both TLR4*^WT^* and TLR4*^mut^* mice infected with VEEV TC-83 compared with uninfected mice. However, the induction of MMP-2 in infected TLR4*^mut^* mice was significantly lower compared with the infected TLR4*^WT^* mice at days 4 and 6 PI (Figure 3b).

Intracellular adhesion molecule 1 (ICAM-1) is constitutively expressed at low levels on the vascular endothelium of the BBB and perivascular astrocytes. However, ICAM-1 is upregulated in pathological conditions in the brain and plays an important role in cell-cell adhesion, extravasation and inflammatory responses [32]. Even though ICAM-1 was significantly induced in the brains of both infected TLR4*^WT^* and TLR4*^mut^* mice at days 4, 6 and 8 PI, it was induced to significantly lower extents in the cortexes of infected TLR4*^mut^* mice (Figure 3c).

Furthermore, C-C motif ligand 2 (CCL-2) was significantly induced in the cortexes of both infected TLR4*^WT^* and TLR4*^mut^* mice. However, CCL-2 was significantly less induced in the TLR4*^mut^* mice compared with the TLR4*^WT^* mice at days 4 and 6 PI, but was induced to similar levels at day 8 PI (Figure 3d). CCL-2 attracts monocytes and T cells as well as contributes to BBB permeability [33]. The reduced inductions of MMP-9, MMP-2, CCL-2 and ICAM-1 in the TLR4*^mut^* mice support the role of TLR4 mediating BBB permeability during VEEV infection.

### Induction of inflammatory cytokines in the brains of mice is not mediated by TLR4 during VEEV TC-83 infection

**Figure 4.**
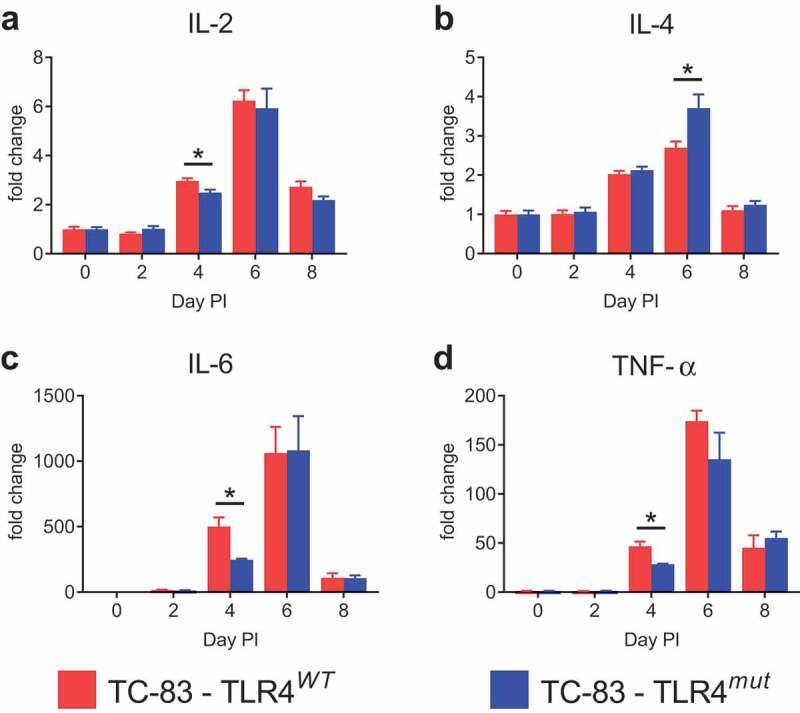
**Induction of IL-2, IL-4, IL-6 and TNF-α in the cortexes of TLR4*^WT^* mice and TLR4*^mut^* mice during VEEV TC-83 infection**. The induction of IL-2 (a), IL-4 (b), IL-6 (c) and TNF-α (d) during VEEV TC-83 infection was assessed in cortical tissue from TLR4*^WT^* mice and TLR4*^mut^* mice. The fold change of these proteins is expressed as the ratio of the amount of protein in infected mice relative to uninfected mice of the same genotype. Mock-infected (PBS-instilled) mice are represented as Day 0 PI. Data are presented as mean ± SEM (*n* = 3–5 mice per group). Statistically significant differences are indicated as *, *p* < 0.05. Statistical significance was evaluated by unpaired two-tailed Student’s t-tests or Mann-Whitney tests

In order to determine the effects of TLR4 signaling in the brain on the inflammatory cytokine response during VEEV TC-83 infection, the levels of interleukin 2 (IL-2), IL-4, IL-6 and tumor necrosis factor alpha (TNF-α) were determined in cortical homogenates from days 2, 4, 6 and 8 PI. VEEV TC-83 infection significantly induced IL-2, IL-4, IL-6 and TNF-α in the brains of both TLR4*^WT^* and TLR4*^mut^* mice (Figure 4). IL-2, IL-6 and TNF-α were all induced to significantly lesser extents in the cortexes of the infected TLR4*^mut^* mice compared with the infected TLR4*^WT^* mice at day 4 PI, but they were induced to similar levels in the brains of both genotypes by VEEV TC-83 infection at days 2, 6 and 8 PI. IL-4 was induced significantly more at day 6 PI in the infected TLR4*^mut^* mice compared with the infected TLR4*^WT^* mice, but there were no other significant differences for IL-4 at any other time point.

### IFN-γ is less induced in the brains of VEEV TC-83-infected TLR4^mut^ mice

**Figure 5.**
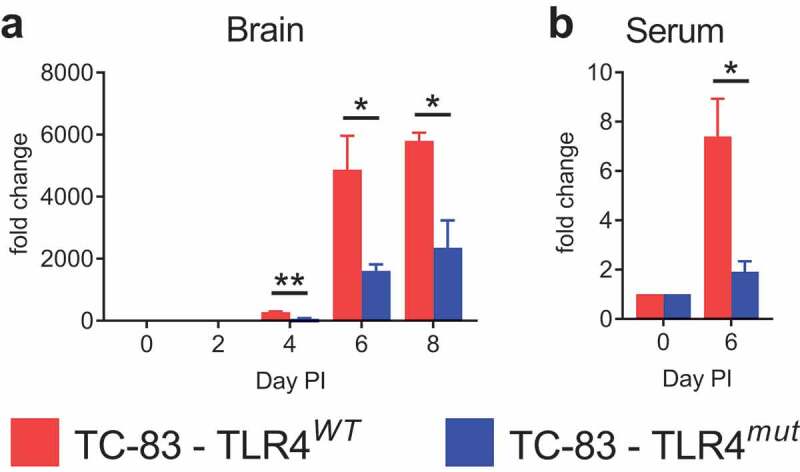
**IFN-γ induction in the cortexes and sera of TLR4*^WT^* mice and TLR4*^mut^* mice during VEEV TC-83 infection**. The induction of IFN-γ in the cortexes (a) and sera (b) during VEEV TC-83 infection was assessed in TLR4*^WT^* mice and TLR4*^mut^* mice. The fold change of IFN-γ is expressed as the ratio of the amount of IFN-γ in infected mice relative to uninfected mice of the same genotype. Mock-infected (PBS-instilled) mice are represented as Day 0 PI. Data are presented as mean ± SEM (*n* = 3–5 mice per group). Statistically significant differences are indicated as *, *p* < 0.05; **, *p* < 0.01. Statistical significance was evaluated by unpaired two-tailed Student’s t-tests or Mann-Whitney tests

Interferon gamma (IFN-γ), the only member of the type II class of IFNs, is a dimerized soluble cytokine that is released predominately by leukocyte subtypes, such as natural killer (NK) cells, Th1 cells and cytotoxic T lymphocytes. Importantly, IFN-γ is also a mediator of BBB permeability during CNS infections [34,35]. There was a significant induction of IFN-γ in the brains of both TLR4*^WT^* and TLR4*^mut^* mice infected with VEEV TC-83. However, IFN-γ was induced to a lesser extent in the cortexes of infected TLR4*^mut^* mice compared with infected TLR4*^WT^* mice at days 4, 6 and 8 PI (Figure 5a). In addition, day 6 PI was the only day with a significant induction of IFN-γ in the serum of infected mice and these levels of induction were significantly less than in the brains of infected TLR4*^mut^* mice compared with infected TLR4*^WT^* mice (Figure 5b). These data demonstrate that defective TLR4 signaling impairs IFN-γ induction in the brains of TC-83-infected mice.

### Loss of TLR4 signaling does not alter the immune population in the brain during TC-83 infection

**Figure 6.**
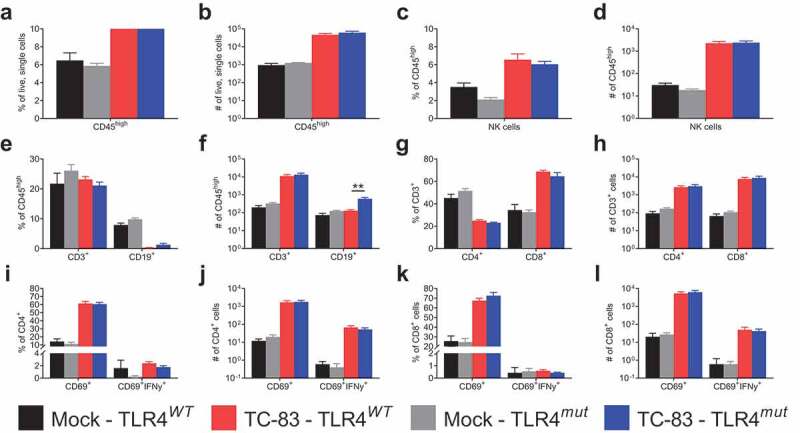
**Immune cell populations in the brains of VEEV TC-83-infected TLR4*^WT^* mice and TLR4*^mut^* mice at day 6 PI**. The immune cell populations in the brains of uninfected and TC-83-infected mice were determined by FACS analysis 6 days after infection. The percentage (a) and number (b) of CD45^hi^ cells and (c-d) NK cells. The percentage (e) and number (f) of CD3^+^ or CD19^+^CD45^hi^ cells. The percentage (g) and number (h) of CD3^+^ cells that were CD4^+^ or CD8^+^. The percentage (i) and number (j) of CD4^+^ cells that were CD69^+^ or CD69^+^IFNγ^+^. The percentage (k) and number (l) of CD8^+^ cells that were CD69^+^ or CD69^+^IFNγ^+^. Data are presented as mean ± SEM (*n* = 5 mice per group). Statistically significant differences are indicated as **, *p* < 0.01. Statistical significance was evaluated by unpaired two-tailed Student’s t-tests or Mann-Whitney tests

To determine if defective TLR4 signaling alters the immune cell population in the brains of TC-83-infected mice, brain homogenates were stained for flow cytometry analysis on day 6 PI when the differences in MMP-9, ICAM-1, CCL-2 and IFN-γ induction were greatest (Figure [3,5]). There were no differences in the numbers or percentages of CD45^hi^ leukocytes (Figure 6(a,b)) or NK cells (Figure 6(c,d)) in the brains of infected TLR4*^WT^* and TLR4*^mut^* mice. There was a significant increase in the number of B cells (CD45^hi^CD19^+^) in the infected TLR4*^mut^* mice compared with the infected TLR4*^WT^* mice despite there being no significant difference in the percentage of CD45^hi^ leukocytes that are B cells (Figure 6(e,f)). However, there were no significant differences in the percentage or number of T cells (CD45^hi^CD3^+^) (Figure 6(e**,f)**), whether CD4^+^ or CD8^+^ (Figure 6(g**,h)**). Furthermore, there were no detectable differences in T cell activation (CD69^+^) or intracellular IFN-γ expression (Figure 6(i**-l)**) between the two mouse substrains.

**Figure 7.**
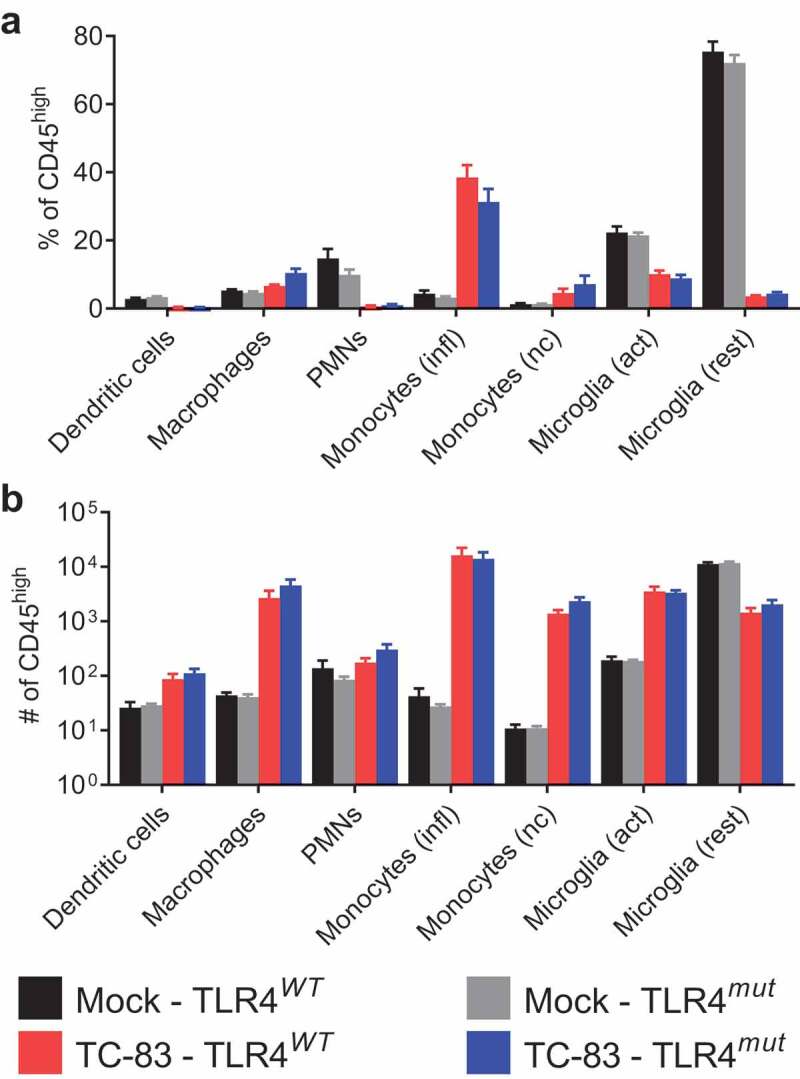
**Myeloid/microglial cell populations in the brains of VEEV TC-83-infected TLR4*^WT^* mice and TLR4*^mut^* mice**. The myeloid/microglial immune populations in the brains of uninfected and TC-83-infected mice were determined by FACS analysis 6 days after infection. The percentage (a) and number (b) of dendritic cells, macrophages, polymorphonuclear neutrophils (PMNs), inflammatory (infl) monocytes, non-classical (nc) monocytes, activated (act) microglia and resting (rest) microglia in the brains. Data are presented as mean ± SEM (*n* = 5 mice per group). Statistical significance was evaluated by unpaired two-tailed Student’s t-tests or Mann-Whitney tests

There were also no significant differences in myeloid/microglial immune populations between infected TLR4*^WT^* and TLR4*^mut^* brains. Analysis included dendritic cells, macrophages, polymorphonuclear neutrophils, inflammatory monocytes, non-classical monocytes, activated microglia and resting microglia (Figure 7(a**,b)**). In addition, there were no preexisting differences in CNS leukocytes populations between the two substrains.

## Discussion

Encephalitis is a serious and potentially fatal outcome of human VEEV infection; however, the mechanisms in the CNS that drive neurologic disease during VEEV infection are ill-defined. This lack of understanding coupled with the absence of approved vaccines for human use and specific therapies for VEEV infection underscores the need for determining the neuropathogenic mechanisms of encephalitic alphaviruses. During VEEV infection in the CNS, the induction of BBB permeability and concomitant MMP-9 upregulation suggests that these events are critical neuropathogenic events. Because of the dependence of MMP-9 induction and BBB permeability during neurologic diseases on TLR4, the role of TLR4 during VEEV TC-83 infection was examined.

Previously, intranasal VEEV TC-83 infection has been shown to cause increased BBB permeability and symptomatic infections in C57BL/6 mice, *Ifit1* knockout mice [9] and TLR4*^WT^* mice [36] with the latter two developing neurological symptoms and mortality. Here, TLR4*^WT^* mice in parallel with TLR4*^mut^* mice were used to determine the effects of TLR4 on the susceptibility of mice to disease as a result of intranasal VEEV TC-83 infection. This model was used because VEEV TC-83 infection in TLR4*^WT^* mice results in increased BBB permeability [36] as does wild-type VEEV infection in mice [12]. In addition, the brain pathology in this model is similar to the brain pathology seen in mice infected with other VEEV strains, including meningoencephalitis, gliosis and multifocal neuropil vacuolation [29,37–39]. However, VEEV TC-83 infection is limited to the brain with minimal spread outside the CNS after aerosol or intranasal exposure, thus limiting the effects of systemic inflammation on the BBB, in contrast to VEEV TrD infection. Therefore, VEEV TC-83 infection in C3H mice remaining relatively restricted to the CNS while still mediating neurologic disease provides a better model, compared with using VEEV TrD, to examine the role of BBB permeability during CNS infection with VEEV and the mechanisms by which increased BBB permeability is induced. Intranasal exposure of TLR4*^WT^* mice and TLR4*^mut^* mice to VEEV TC-83 demonstrate that TLR4*^mut^* mice are less susceptible to disease and that TLR4 plays a role in mediating this disease. The difference in susceptibility of TLR4*^mut^* mice to disease caused by VEEV TC-83 infection was not because of a decrease in viral replication in the brain. Viral titers were decreased in the cortexes of infected TLR4*^mut^* mice compared with infected TLR4*^WT^* mice at days 1 and 6 PI and increased at days 2 and 8 PI suggesting there might be slight differences in the kinetics of virus replication in the brains of these mice. However, these differences were not statistically significant suggesting that there are other factors associated with increased survival of TLR4*^mut^* mice.

One potential mechanism of the decreased VEEV-related disease that we observed in TLR4*^mut^* mice as compared to wild-type mice is the reduced induction of BBB permeability as demonstrated by NaF and DEX-680 extravasation. Our data are consistent with the findings of a previous study, which showed that inhibiting the induction of BBB permeability with a broad spectrum MMP inhibitor, GM6001, delayed the onset of disease and decreased mortality in mice infected with wild-type VEEV [12]. However, our study expands on earlier work by demonstrating that upregulation of MMP-9 and the induction of BBB permeability are dependent on TLR4 during VEEV TC-83 infection in mice. The induction of BBB permeability and MMP-9 to lesser extents in infected TLR4*^mut^* mice compared with infected TLR4*^WT^* mice and the relative increased induction of MMP-9 compared with MMP-2 in infected TLR4*^WT^* mice suggest that MMP-9 is the major mediator of increased BBB permeability during VEEV infection. During ischemic stroke in mice, MMP-2 and MMP-9 have different roles in BBB disruption where MMP-2 does not contribute to acute tissue damage as evidenced by the absence of neuroprotection of MMP-2 knockouts [40]. In contrast, MMP-9 knockout provides strong protection in ischemic stroke mouse models [17]. Additional evidence comes from studies in which MMP-9, but not MMP-2, is neurotoxic in hippocampal slice preparations [41] and from work showing that MMP-9 knockout mice are less susceptible to disease caused by peripheral WNV exposure compared with wild-type mice [14]. Therefore, our results and corroborating studies in other systems indicate that MMP-9 is a critical mediator of BBB permeability and, potentially, neuronal damage during CNS infections with VEEV.

In addition to the lack of significant MMP-9 induction, the lesser inductions of ICAM-1, CCL-2, IFN-γ and BBB permeability in infected TLR4*^mut^* mice suggested there could be a difference in the population of infiltrating immune cells in the brain. NK cells were of particular interest because they release IFN-γ and have been suggested to have a role in mediating VEEV TC-83 pathogenesis in C3H/HeN mice [30]. Not only were there no differences in NK cell number in the brains of the two infected substrains, there were no differences in other immune cell populations examined with the exception of more B cells in the infected TLR4*^mut^* mice. In both TLR4*^WT^* and TLR4*^mut^* mice, there was still a significant induction of BBB permeability, ICAM-1, MMP-2 and CCL-2 in the brains during VEEV TC-83 infection. Therefore, the induction of these factors suggests that both the TLR4*^WT^* and TLR4*^mut^* mice have similar immune responses in the brain at day 6 PI. Additional studies to include more time points and functional assays will have to be performed to determine if the phenotypes of infiltrating immune cells are altered as a result of defective TLR4 signaling.

The combination of impaired TLR4 signaling in the TLR4*^mut^* mice with significantly less IFN-γ induction in the brains suggests that these may play a role in neurotoxicity and inflammation-induced neurodegeneration. A study using organotypic hippocampal slice cultures from rats in the absence of infiltrating leukocytes found that activating TLR4 with LPS induced the stable release of proinflammatory cytokines (IL-1β, TNF-α and IL-6; IFN-γ was not significantly induced) while IFN-γ did not. Additionally, exposure to LPS or IFN-γ alone resulted in released nitric oxide (NO) through upregulated inducible nitric oxide synthase (iNOS). However, LPS or IFN-γ exposure alone only moderately affected intrinsic neuronal excitability and gamma oscillations. Strikingly though, co-exposure to LPS and IFN-γ resulted in a higher magnitude of NO release and iNOS expression as well as massive neuronal dysfunction and death to the point that the slice cultures disintegrated and neuronal activity was completely absent. These studies showed that TLR4-activated microglia require concomitant IFN-γ signaling to induce massive inflammatory neurodegeneration [42]. In addition, iNOS knockout mice infected with wild-type VEEV have increased survival times [39], but these mice are still susceptible to LPS-induced death [43]. Therefore, the lack of mortality and disease in the TC-83-infected TLR4*^mut^* mice suggests that VEEV infection may require the coactivation of TLR4 and IFN-γ receptors to mediate neurologic disease, potentially independent of iNOS, despite the presence of infiltrating leukocytes and pro-inflammatory cytokines. Future studies with VEEV will examine the co-activation of these receptors on NO release and neurodegeneration both *in situ* and *in vivo*.

Excessive inflammatory responses are present in severe diseases caused by a wide variety of viruses, including respiratory syncytial virus (RSV) [44], influenza A virus (IAV) [45], Ebola virus (EBOV) [46], dengue virus (DENV) [47] and severe acute respiratory syndrome coronavirus [48]. The role of TLR4 in the pathogenesis of some of these viral diseases is demonstrated by the reduced induction of cytokines and chemokines as a result of treatment with TLR4 antagonists [49–53]. Furthermore, treatment with these TLR4 antagonists prevented lethal EBOV [53] and IAV infection [50–52] in mice demonstrating the therapeutic potential of inhibiting TLR4 during viral infections. However, TLR4 knockout mice have either similar survival rates or more severe disease than wild-type mice when infected with EBOV [53] and, in some cases, IAV [54] suggesting that some degree of TLR4 signaling is required for the protective response of TLR4 antagonists against these viruses. Therefore, TLR4 is an attractive target for the development of therapeutics against these viruses and VEEV.

VEEV could be mediating TLR4 activation through one of the viral proteins, damage-associated molecular patterns (DAMPS), or both. The RSV fusion protein [55], EBOV glycoprotein [56,57] and DENV nonstructural protein 1 [49] all activate TLR4. In contrast with these viruses, IAV activates TLR4 by host DAMPs, including HMGB1 [51] and oxidized phospholipids [58]. Intracranial inoculation of VEEV replicon particles into the brains of mice induce BBB permeability, but UV-inactivated VEEV replicon particles do not, suggesting that viral RNA replication is required for BBB permeability induction [59]. This lack of BBB permeability induction by UV-inactivated VEEV replicon particles and the dependence of BBB permeability induction on TLR4 suggests that if a viral protein is activating TLR4, it is either a nonstructural protein or virus replication is needed to produce a sufficient amount of VEEV glycoproteins to activate TLR4 to mediate BBB permeability. In addition, intranasal inoculation of VEEV replicon particles mediating BBB permeability in the cortex despite being restricted to the olfactory bulb [12] suggests that there is a signal that is disseminated through the brain.

Potential mediators of TLR4 activation include HMGB1 and other DAMPs. HMGB1 is a non-histone nuclear protein that can function as a pathogenic inflammatory response in a number of conditions in the brain, including epilepsy [60], septic shock [61], ischemia [62], traumatic brain injury [63], Parkinson’s disease [64], Alzheimer’s disease [65] and multiple sclerosis [66]. After neuronal injury, significant amounts of HMGB1 are passively released from the nucleus and into the extracellular space [67]. In addition, HMGB1 is actively secreted by neurons and glia upon inflammasome activation in neuroinflammatory conditions. In the extracellular space, HMGB1 can alter synaptic function in the brain and mediate inflammation [68]. Furthermore, HMGB1 has been shown to activate TLR4 to induce MMP-9 upregulation following cerebral ischemia [25]. Therefore, the decrease in disease of the TLR4*^mut^* mice during VEEV TC-83 infection might in part be mediated by a decrease in HMGB1 driven pro-inflammatory effects, although this remains to be experimentally demonstrated.

One limitation of these studies is that the substrains of mice were purchased from different vendors, which could have confounding effects on the results of the experiments. For example, there are significant differences in the bacterial and viral components of the gut microbiome between the same strain of mice purchased from different vendors [69]. Additional confounding effects could result from potentially different diets fed by different vendors, the effects of potentially different maternal behaviors of the substrains and potential genetic drift of the substrains. These potential confounds support the need to perform these studies with WT VEEV in WT C57BL/6 mice and TLR4 knockout mice on the same genetic background from the same vendor. However, the established role of TLR4 in regulating MMP-9, which are also demonstrated in these studies, supports the results from the present studies.

In summary, our findings demonstrate that TLR4 is an important mediator of disease pathogenesis in C3H mice infected with VEEV TC-83 as TLR4*^mut^* mice are less susceptible to disease during VEEV TC-83 infection. In addition, the induction of BBB permeability and upregulation of MMP-9 during CNS VEEV TC-83 infection are dependent on TLR4. However, because induction of BBB is a relatively late and critical event in viral encephalitis, TLR4 may be a potential target for the development of therapeutics for encephalitic alphaviruses. Further studies are needed to understand the precise mechanisms by which TLR4 mediates encephalitic disease during VEEV infection.
